# Psychometric properties of the Persian version of abridged Connor-Davidson Resilience Scale 10 (CD-RISC-10) among older adults

**DOI:** 10.1186/s12888-022-04138-0

**Published:** 2022-07-23

**Authors:** Hassan Rezaeipandari, Asghar Mohammadpoorasl, Mohammad Ali Morowatisharifabad, Abdolreza Shaghaghi

**Affiliations:** 1 Department of Aging Health, School of Public Health, Shahid Sadoughi University of Medical Sciences, Yazd, Iran; 2 Elderly Health Research Center, School of Public Health, Shahid Sadoughi University of Medical Sciences, Yazd, Iran; 3 Health and Environment Research Center, Tabriz University of Medical Sciences, Tabriz, Iran; 4 Department of Health Education and Promotion, Faculty of Health Sciences, Tabriz University of Medical Sciences, Tabriz, Iran

**Keywords:** Resilience, Older adults, Psychometric properties, CD-RISC

## Abstract

**Background:**

Resilience is an ability of an individual to respond positively to environmental challenges. This ability could help elderly people to better cope with their age-related changes and diseases. The aim of this study was to examine the psychometric properties of Persian version of abridged Connor- Davidson scale of resilience among Iranian elderly people with chronic diseases.

**Methods:**

Standard translation/back-translation procedure was applied to prepare the Persian version of abridged Connor-Davidson scale of resilience (CD-RISC 10-P) and its face and content validity were examined by an expert panel. The internal consistency and reliability of the drafted CD-RISC 10-P were investigated using the Cronbach’s alpha and intra-class correlation coefficients. A sample of 400 Muslim and Zoroastrian Persian older adults residing in the city of Yazd, Iran was recruited to assess factor structure of CD-RISC 10-P using the confirmatory factor analysis.

**Results:**

The calculated values of the Cronbach’s alpha (0.89) and ICC (0.90) coefficients were in the within of acceptable range. The confirmatory factor analysis outputs also confirmed the unidimensionality of the CD-RISC 10-P (RMSEA = 0.073, SRMR = 0.030).

**Conclusions:**

The study findings showed that the CD-RISC 10-P is a valid and reliable scale to measure resilience with age-related challenges of chronic diseases among Persian-speaking elderly people. Cross-cultural adaptability of the CD-RISC 10-P is recommended to be assessed in different subgroups of the Iranian elderly people and possibly in other Persian-speaking populations of different countries.

## Background

People of old age are facing with an accumulating trajectory of chronic diseases that raise daily life stresses [1]. Therefore, interventions to enhance their resilience to withstand adversity and bounce back from difficult life events is enormously pivotal for maintaining elders’ mental and emotional health [1]. Connor & Davidson referred to resilience as a personal capability that enables an individual to tap into their strengths for thriving and overcoming outstanding life challenges [2].

Resilience involves an individual’s ability to respond positively to environmental challenges that might be physiological, psychological or social [3]. The concept of resilience does not imply a passive acceptance of damage or threatening situations nor to adopt an unsatisfactory orientation when facing dangerous situations. It is in contrast an individual’s ability to establish bio-psychological balance under difficult conditions [2]. Resilience is a multidimensional concept that includes a set of behaviors and attitudes that empowers individuals to deal with acute and chronic stressful life events [4].

Resilience encompasses physical, psychological and social dimensions in its general sense that represents its multidimensionality. Empirical evidence suggests positive impact of high resilience in reducing depression, other morbidities and also improving quality of life [5]. Therefore, the power of resilience is regarded as an important feature of successful aging [6] while its attributes and possible changes over years still needs to be further speculated.

It has been suggested that aging may be associated with increased resilience due to being more experienced in handling life challenges. This means that older people may be more resilient and better able to cope with the challenges of illnesses and disabilities they are experiencing in their life in comparison to younger age subgroups [7]. However, diversity of challenges elderly people experience in their daily life such as chronic diseases, stress, retirement, death of spouse, etc. and also inherent discrepancy of contextual social circumstances (e.g. socio-economic and cultural variations, quality and accessibility of social support services etc.) make deep understanding of the phenomenon in different subgroups of elderly people a high priority. This could add to credibility of initiatives that seek identification of the major determinants of resilience in different population samples and its enhancement thereafter [3]. A number of measurement scales have been introduced for application in studies on resilience such as the Resilience Scale for Adults (RSA) [8], the Brief Resilience Scale (BRS) [9], the Resilience Scale (RS) [10] but there are reports about limitations of these measures in terms of wider acceptability and applicability [11]. The Connor- Davidson scale of resilience (CD-RISC) is a self-report scale comprised of 25 items that aims to measure resilience among the potential respondents [2]. Its psychometric properties were assessed in several studies [12–15] and seems to be a reliable measurement tool for its intended purpose.

Due to observed instability of the five-factor structure the original CD-RISC 25 and inability of the researchers to agree on its best possible factor compositions [11], Campbell-Sills & Stein [16], extracted and validated a summarized version of the CD-RISC with 10 items (CD-RISC 10) that indicated robust loading values and high level of consistency when compared to the original 25 items CD- RISC. Validity and reliability of the two versions have been studied in several studies [17–19] and both (CD-RISC 25 and CD-RISC 10) revealed good psychometric properties. However, CD-RISC 10 possessed better and more established factor structure and is suggested to be more robust, efficient, simple to use and parsimonious [11].

The CD-RISC 10 was used to measure resilience among elderly people [3] in the past but distinct socio-cultural attributes may render different results in diverse sample of elderly populations [20]. Since, there are no validated tools to study resilience among Persian-speaking aged populations, this study was aims to examine psychometric properties (reliability, validity and cultural adaptability) of the CD-RISC 10 for its possible utilization among Persian-speaking elderly populations.

## Methods

### Study sample

A total of 400 older adults (200 Zoroastrian and 200 Muslim individuals) aged 60 years old and above were recruited from two primary health care centers in the city of Yazd, the center of Iran. Since, one of the objectives in this study was to compare resilience capacities between the two religious subgroups of the Iranian elderly people (Zoroastrians and Muslims) and to ensure diversity of the selected sample, inclusion of participants from both mentioned religious affinities was decided. The sample size was based on the recommended sample size (8 participants per item) for factor analysis by the MacCallum et al. and Boomsma [21, 22]. According to the suggestion different subsamples are required to perform the exploratory and confirmatory factor analyses (EFA, CFA). To ensure adequacy of the sample size and robustness of the yielded outputs the number of participants was doubled for each sub-sample.

For selection of participants from two health care centers, convenience sampling method was used. Then the participants were randomly selected from the Iranian national health system’s database (SIB) and they were invited to participate in the study.

The study participants were residents of the city of Yazd in the age of 60 years and above and also were diagnosed with at least one to three simulataneous chronic diseases. Main aim of selecting eldery people with at least a chronic disorder was to have a sample with greatest accuracy and resembalance to the original and ordinary population of the country’s eldery population. Other inclusion criteria included ability to speak and understand Persian language, not having considerable hearing loss to make inerviews challenging and also not having a major cognitive problems which was tested based on the Abbreviated Mental Test Score (AMTS) [23]. People with severe medical conditions or cognitive impairment that make participation in the study medically inconsistent or to make a respondent cognitively dependent on others were excluded to ensure that all participants could follow the study protocol, and assure accuracy of the responses to the survey questions.

### Instrument

The original Connor-Davidson Resilience Scale 10 (CD-RISC 10) [16] that was prepared based on the CD-RISC 25 [2], comprises 10 self-report items, each rated on a Likert-type scale from 0 (not true at all) to 4 (true nearly all the time). In its original version, all of the 10 items load on a single dimension. The total score could range from 0 to 40 with higher scores indicating greater resilience of the respondent. A Persian translated version of the CD-RISC 10 that had been psychometrically tested before on a group of undergraduate nursing students [24] was used as a preliminary draft in this study but to ensure rigor in application of the translated version we performed standard back-translation procedure on the original CD-RISC 10 [16].

### Translation procedure

The English version of the CD-RISC 10 scale [16] was translated into Persian by two academic translators fluent in both English and Persian. In the next stage, the drafted Persian version of the scale was back- translated into English by a proficient translator. Later, this back-translated version was compared with the original English version and minor revisions were made to correct mismatches between the two versions. The prepared Persian version of the CD-RISC 10 [16] was also contrasted with the preliminary Persian draft [24] and all mismatches resolved with consensus. The final adapted Persian version was used for quantitative validity and reliability appraisal. Permission was obtained from the CD-RISC 10 developer (Professor Jonathan RT Davidson) to use the scale and report the study findings.

### Qualitative validity assessment

The CD-RISC 10-P was sent to a panel of experts consisting of fifteen gerontologists, geriatricians, health education and promotion specialists, psychologists and nursing specialists to assess its simplicity of wording, clarity of sentences and concept transferability. Based on the feedback received from the panelists the Content validity index (CVI) and content validity ratio (CVR) were calculated to ascertain content validity of the instrument. A CVI of 0.8 or greater [25] and a CVR of greater than 0.49 [26] were deemed to be in acceptable range. The Persian version of the CD-RISC 10 was also pilot tested on 28 older adults with regard to understandability of the questions and response items. There was a two-week time interval between testing in pilot section.

### Data collection procedures

Data collection was performed by two trained and qualified interviewers through face-to-face interviews (self-completion of the scale was ruled out due to feasibility concerns including the required literacy level) by the registered Zoroastrian and Muslim older adults in the two purposefully selected urban health care centers. The purpose and voluntariness of participation in the study was explained before the interviews’ initiation and written informed consent obtained from the interviewees or their legal guardians (in the case of illiterate individuals) after explanation of the study objectives, procedures and right to withdraw from the study at any time and by any reason without obligation to expose any reason(s). The interviews were started with asking questions about the respondents’ demographic characteristics (including: age, gender, marital status, educational level, living status and chronic diseases) and CD-RISC 10-P items were impugned later.

### Quantitative validity assessment

The CD-RISC 10-P scores’ distribution for possible skewness or kurtosis by conducting a graphic analysis and statistical test (Kolmogorov–Smirnov test) [27] and also the ceiling and floor effects were checked by examining the percentage of respondents with very low or very high scores defined on the base of the core range (< 15%) [28].

The internal consistency and test-retest reliability of the CD-RISC 10-P were assessed using the Cronbach’s alpha and Intra-Class Correlation (ICC) coefficients.

The values of Cronbach’s Alpha Coefficient greater than 0.8 and ICC greater than 0.9 were considered to be in acceptable range [29, 30].

Confirmatory factor analysis (CFA) was performed to verify unidimensionality of the scale as observed in the original study [16] and other psychometric assessments of the scale [17, 18]. According to the Hu and Bentler’s suggestion the value of Root Mean Square Error of Approximation (RMSEA) below 0.05 can be deemed as acceptable [31].

The value of Standardized Root Mean Square Residual (SRMR) smaller than 0.05 was considered acceptable [32]. The Comparative Fit Index (CFI) and Tucker Lewis index (TLI) values > 0.90 were considered to represent acceptable model fit, and values above 0.95 as very good fit to the study data [31]. The IBM SPSS version 24 [33], and STATA version 14 [34] were used for data analysis.

## Results

### Participants characteristics

The mean age of the recruited older adults was 70.63 (SD = 8). Of them, 41.8% were male. Among the study participants 72% were married and 17.7% were illiterate. The most reported chronic diseases by the study respondents included hypertension (62.5%), arthritis (59.3%), hyperlipidemia (44.3%) and diabetes (40.3%). The mean resilience score of the respondents was 19.76 ± 8.83 and the approached male interviewees indicated a better resilience capacity (21.08) than their female counterparts (18.82) (*P* = 0.02, df = 398).

### Content validity

The values of CVR and CVI for scale were 0.85 and 0.90, respectively that were in acceptable range [29]. Findings of the scale’s pilot testing on 28 older adults yielded to minor changes in terms of wordings employed.

### Reliability

The calculated ICC coefficient over two-week time interval were acceptable in the Muslim (0.91), Zoroastrian (0.90) subgroups, and in the total sample (0.90). Also, value of the Cronbach’s alpha in the Muslim (0.89) and Zoroastrian (0.90) subgroups were in the acceptable range [27].

### Feasibility

The estimated ceiling and floor effects for overall score of the CD-RISC 10-P scale and also in Muslims and Zoroastrian participants were presented in Table 1. Ceiling or floor effects are not observed in the data that represents measurement accuracy of the output variable [28]. The values of skewness and kurtosis test for examination of the data normality were reported in Table 1. As indicated they have verified symmetry and normal distribution of the collected data [27]. Missing data was not detected for the observations and the mean score of resilience among Zoroastrians older adults was higher than Muslims.

**Table 1:** The Resilience scores of the study participants in the cross-cultural adaptation and psychometric validation of the Persian version of the Connor-Davidson Resilience Scale 10 (CD-RISC 10-P)

**Studied samples**		**N**	**Min**		**Max**		**Mean**	**SD**		**Skewness**		**Kurtosis**		**Floor (%)**		**Ceiling (%)**	
**Muslims**		200	3		40		16.96	7.85		0.70		-0.14		0.5		0.5	
**Zoroastrians**		200	0		40		22.57	8.89		-0.21		-0.51		0.5		1.0	
**Total**		400	0		40		19.76	8.83		0.24		-0.75		0.3		0.8	

### Construct validity

To quantify the data from the individual items on the questionnaire, a factor analysis was performed. The minimum factor loading for the Q1 (able to adapt to change) were 0.70, 0.62 and 0.61 in the two studied groups (Muslims, Zoroastrians) and in total, respectively. Additionally, except for a few items, most of factor loadings exceeded 0.70 which revealed the model's acceptable validity (Table 2).

The calculated regression weights for the scale’s items that represent direction and magnitude of the parameters’ estimates were positive, in the range of 0.63–0.74 and statistically significant (*P* < 0.05). These findings further support construct validity of the CD-RISC 10-P.

**Table 2:** Factor loadings of the  items in the Persian version of Connor-Davidson Resilience Scale (CD-RISC 10-P) according to the confirmatory factor analysis outputs

Groups Items	Muslims	Zoroastrians	Total
**Q1**	0.70	0.62	0.61
**Q2**	0.72	0.66	0.65
**Q3**	0.79	0.70	0.69
**Q4**	0.71	0.72	0.70
**Q5**	0.72	0.71	0.70
**Q6**	0.71	0.67	0.67
**Q7**	0.76	0.73	0.73
**Q8**	0.70	0.72	0.75
**Q9**	0.76	0.63	0.66
**Q10**	0.76	0.75	0.77

The CFA results revealed an adequate unidimensional factor model [31] that consists with the original CD-RISC 10 [16] as presented in Table 3. The estimated values of the goodness-of-fit indices in both sub-groups of the studied Muslims and Zoroastrian older adults were in acceptable range and no considerable variations were observed between the groups. The χ^2^ value was also significant and the estimated RMSEA in this study was higher than 0.05. It is suggested that the obtained RMSEA values between 0 and 0.05 indicate good fit and 0.05 to 0.08 indicate acceptable fit [35].

**Table 3:** The estimated model fit indices according to the confirmatory factor analysis of the structure of the Persian version of the CD-RISC 10 (*n* = 400)

Groups	χ2	df	CFI	TLI	RMSEA	SRMR
**Muslims**	33.85	24	0.980	0.963	0.064	0.048
**Zoroastrians**	33.89	26	0.985	0.973	0.055	0.042
**Total**	74.55	25	0.976	0.955	0.073	0.030

## Discussion

The common pattern of comorbidities in later years of life and consequent hassles that they pose on elderly people make resilience a tangible trait to confront resulted infirmities [36]. Studies have examined psychometric properties of the resilience tool (CD-RISC 25) in Iran [37, 38], but no study has been found among the older adults to use CD-RISC 10. The main aim of this study was to evaluate the psychometric attributes of abridged CD-RISC 10 for use on Persian-speaking older adults.

The study findings indicated good internal consistency and test-retest reliability (ICC) of the CD-RISC 10-P and unidimensionality of the scale as observed in the original study [16]. The estimated Cronbach’s alpha coefficient in this study was congruent with the observed measure of internal consistency of the CD-RISC 10 in other studies [36, 39–43] but higher than the calculated values in the original study [16] and other studies [18, 19, 44, 45]. The study results also showed acceptable goodness-of-fit indices which represent applicability of the CD-RISC 10-P in the Persian speaking Zoroastrian and Muslim older adults.

Application of the original English version [16] of the CD-RISC 10 and other psychometric studies that tested its appropriateness for use in different sub-samples i.e. Australian cricketers [17], women with breast cancer [18], Chinese undergraduates and depressive patients [39], patients with fibromyalgia [40], young adults [44], vulnerable Colombian adolescents [46] people with lower-limb amputation [47], Greek adults of the general population [48], Russian youth sample [49] and low-income African American men [50] all represented unidimentionality of the instrument. But EFA and CFA outputs in the study of Aloba et al. yielded a two-factor model fit with the data collected from student nurses in Southwestern Nigeria [19]. The discrepancy might be related to differences in the design of studies, study samples and use of different data collection methods (i.e. cross-sectional versus cohort, inclusion of different age-groups in study sample, self-completion versus performing interview) than a real mismatches among the findings. Conforming psychometric attributes of the CD-RISC 10-P with observed findings in the studies on samples from different sub-cultures [36, 42, 45, 51] might reflect its cross-cultural adaptability [40] that need to be further investigated in future studies on other sub-samples and cross-border Persian speaking older adults. Simplicity and its convenient usability among older adults due to limited number of items makes CD-RISC 10-P and ideal choice for utilization in studies on capacities of elderly people to cope with the adverse consequences of their generally occurring chronic illnesses.

## Conclusions

This study indicated that the Persian version of the CD-RISC 10 could be considered as a valid and reliable scale for measuring resilience among Persian speaking older adults in research or in clinical practice and health care settings after its further psychometric assessment in different cross-cultural and socio-economically diverse subgroups of Persian-speaking elderly people.

### Study limitations

This study has several limitations therefore; the findings must be interpreted with caution. Different socio-economic background of the study participants might cast a shadow on the estimated resilience scores of the participants. There is also possibility that economic and political turbulences in Iran have posed an effect on the respondents’ answers to questions about their ability to cope and resilience capacity when confronting with diseases (due to repeated and accumulated hits from multiple stressors that can alleviate resilience capacities of the individuals). Thus the observed scores of resilience might reflect the recruited people capabilities in exceptional circumstances than their authentic baseline capacity to deal with dilemmas of chronic diseases in a normal condition. This is one the probable confounders in this and similar studies on resilience capacities of people that should carefully be considered in future studies. Those people who were under multiple stressors for a relatively long time (or chronically) might give contrasting responses to questions about their resilience capacities than people who experienced a normal life with conventional lifetime stresses. Literally, individuals’ personal and social alostatic load could have impact on their perceptions about their resilience capacities. Caution must be taken in interpretation of study findings based on the mainstream social/economic conditions in location where resilience studies are taking place.

The CD-RISC 10-P is a self-reported scale and a common methodological restrain exist in using this kind of data collection method in studies [52]. The study participants due to reasons such as having reservation in responding to questions by an outsider might not be willing to expose their real experience or thoughts (e.g. in response to questions such as: “*I can deal with whatever comes my way*”, “*I believe I can achieve my goals, even if there are obstacles*”, “*I am able to handle unpleasant or painful feelings like sadness, fear, and anger*” [2]. This could be especially true in working with ethnic or religious minorities. Recall bias could be other potential source of bias in this study considering the age range of the study sample that prone them to misrepresentation of their actual experience in life or giving inaccurate responses to questions about their personal capabilities or limitations.

Due to lack of a criterion measure and also logistic, budgetary and administrative challenges predictive and convergent validity of the CD-RISC 10-P were not examined in this study. Therefore, it was not possible to determine degree of agreement or inconsistency between the applied instrument’s findings with possible obtainable results in application of an instrument that conceptually measure for similar or fully dissimilar underlying constructs.

An official Persian version of the CD-RISC 10 was available by the original CD-RISC 10 developers since 2015 but the authors were not aware of its presence in the time of data collection stage of this study (Jan-June 2019). The prepared version of the CD-RISC 10 in this study have almost identical structure with the official version and only very slightly was modified for use among elderly people.

Therefore, further scrutiny is warranted in comparison of the study findings with results of other studies that used different version of the Persian translated CD-RISC 10.

For all these reasons generalizability of the study findings may be limited and they warrant further scrutiny in future studies.

## Data Availability

The datasets generated and/or analysed during the current study are not publicly available due to the confidentiality agreements with the institutional level Medical Ethics Board of Trustees (MEBoT) but can only be made available to bona fide researchers from the corresponding author on reasonable request subject to a non-disclosure agreement.
